# Venereal Memoranda

**Published:** 1886-08

**Authors:** 


4. VENEREAL MEMORANDA; *a Manual for the Student and Practitioner. By* P. A. MORROW, A. M., M. D., *Clinical Professor of Venereal Diseases, etc. New York:* WILLIAM WOOD & CO. *1885.*

1. The fourth edition of the manual written by Messrs. Hill & Cooper, has all the merits of those which preceded. It is, like many of its fellows, defective in the important matters on which it does not and cannot touch ; but this is true of all hand-books concisely written. For example, the important subject of chronic prostatitis is here considered in four lines ; and, if the student who purchases the volume should be confronted with a case of this character and should desire to know how to make a differential diagnosis between chronic prostatitis of blennorrhagic origin, and one of the forms of the too common tubercular affection of the prostate, he would have to look elsewhere for his facts. All said, however, the manual is an excellent guide to the student in the field which it concisely covers.

2. Squire's little volume comes to us with the musty aroma of a past generation of dermatologists ; and is an interesting souvenir for the collector of dermatological bric-a-brac of the day when Willan taught how to classify diseases of the skin by the external lesion. For example, under the heading "bullæ," we find discussed "pemphicus and rupia!" Can we trust our eyes ? Is this the day when at least two different forms of bullous exanthem have been shown to originate from ingestion of the iodine compounds alone ? Have we been sleeping like our friend Rip Van Winkle, and is this the rusty flint-lock that lay at our side when we went up the mountains twenty years ago ? This is not a laughing matter ; it is one of the most interesting little souvenirs of the way dermatology was taught years ago that one can pick up for its price.

3. Piffard's work in its third edition, with all the author's odd opinions on "rheumides" and the like, is far in advance of the Englishman's miniature book. It is well known, and deserves the estimate that the young gentlemen place upon it when they study the cases in the clinic. It is, however, surpassed by Duhring's little manual.

4. Morrow's little companion to Piffard's manual is really well and concisely written, and bears evidence of having been thoughtfully prepared. It is epigrammatic in its terseness, accurate in its statements, and useful in many ways to the beginner; scarcely to others. There are objectionable features which may be pardoned in the general excellence of the book. For example, when we read, that "a prostitute is safer at night than in the morning, when contagious secretions have had time to accumulate," we feel like entering a protest against all such literature. Far better direct men how to light cigars in the vicinity of nitro-glycerine. Dr. Morrow retains the nomenclature which includes such terms as "ecthyma-form syphilide," "impetigo-form syphilide," etc. We have before this protested against describing one disease in terms of another. When the student reads of a "variola-form syphilide," he wonders what variola looks like; and, if advanced enough to know, he naturally asks, "in what stage of the evolution of the variolous papulo-vesiculo-pustule does it resemble the special syphiloderm here described?" better go back to the fathers of medicine, who called diseases of the skin by names which children could understand, such as "honey," "wolf," pig, etc.

